# Influenza A Virus and Influenza B Virus Can Induce Apoptosis via Intrinsic or Extrinsic Pathways and Also via NF-*κ*B in a Time and Dose Dependent Manner

**DOI:** 10.1155/2016/1738237

**Published:** 2016-03-02

**Authors:** Ibrahim El-Sayed, Khalid Bassiouny, Aziz Nokaly, Ahmed S. Abdelghani, Wael Roshdy

**Affiliations:** ^1^Genetic Engineering and Biotechnology Research Institute (GEBRI), University of Sadat City, Sadat City 32897, Egypt; ^2^Faculty of Medicine, Al-Azhar University, Cairo 35527, Egypt; ^3^Central Public Health Laboratories (CPHL), Cairo 11613, Egypt

## Abstract

Influenza viruses are able to cause annual epidemics and pandemics due to their mutation rates and reassortment capabilities leading to antigenic shifts and drifts. To identify host response to influenza A and B viruses on A549 and MDCK II cells at low and high MOIs, expressions of MxA and caspases 3, 8, and 9 and BAD, TNF*α*, and I*κ*B*α* genes were measured in the cells supernatants. H1N1 and H3N2 prefer to initially enhance the intrinsic pathway, determined by higher caspase 9 activity in MDCK II cells compared to caspase 8 activity and vice versa in A549 cells at different MOIs, while INF B prefers extrinsic pathway in A549 cells according to significant low or undetectable caspase 9 activity and high activity of caspase 8 but also can induce intrinsic pathway in MDCK II cells as determined by significant low or undetectable activity of caspase 8 and high caspase 9 activity at different MOIs; the considerable MxA expression was found in influenza A and B viruses infected A549 and MDCK II cells at low MOIs. In conclusion, influenza A and B viruses induced extrinsic and intrinsic apoptosis in parallel, and the induction was associated with viral infection in a dose dependent manner.

## 1. Introduction

Influenza A virus, a major cause of morbidity and mortality in humans, is primarily a pathogen of the upper respiratory tract; its infection results in both respiratory effects and constitutional effects [1, 2].

Influenza viruses A and B infection induces distinct apoptosis profiles; the differential biological effects of the influenza A and B viruses have been the focus of intense research [3].

Influenza viruses are able to cause annual epidemics and pandemics due to their mutation rates and reassortment capabilities leading to antigenic drifts and antigenic shifts [4–6].

Influenza viruses belong to the Orthomyxoviridae family and are grouped into types (and subtypes), of which type A and B are the most relevant to humans [7, 8].

They are enveloped, negative single stranded RNA viruses with a segmented genome divided into 8 genes that code for 11 proteins [6] that not only act as viral components but also interact with the pathways of host infected cells, mainly to counteract the antiviral cell response and help the viral replication [9–11]. To date, up to 1023 interactions between viral and host proteins have already been described [6, 9]. Apoptosis induced during influenza virus infection is a major contributing factor to cell death and tissue damage [12–15].

All of the mammalian, as well as all of the avian, influenza viruses tested induce apoptosis in MDCK cells, which prove that apoptosis is a general mechanism by which influenza viruses kill cells and, therefore, that these viruses can be blocked by cellular inhibitors of apoptosis [12].

Studies with the 1918 pandemic virus in macaques showed that activation of the apoptotic pathway was a source of tissue damage during infection [16–18].

In mammalian cells, the apoptotic pathway can be divided into two signaling cascades: the extrinsic and the intrinsic apoptotic pathways [19].

The intrinsic apoptotic pathway acts through the mitochondria upon activation, and this signaling process is highly regulated by the Bcl-2 family of proteins, which consists of both antiapoptotic and proapoptotic members that form a critical decision point within a common cell death signaling pathway [20].

The delicate balance between antiapoptotic and proapoptotic protein activities dictates whether a cell will succumb to an apoptotic stimulus or not [21, 22].

Despite the increasing knowledge in the influenza virus host interactions, most of the published work focuses on influenza A viruses, leaving a gap with respect to influenza B virus host interactions [5, 23].

H3N2 viruses with high NA activities induced high levels of apoptosis (83–94%) and infected 91–98% of cells, while H1N1 viruses with low NA activities were poor apoptosis inducers (11–19%) and infected few (15–21%) cells. The differences in % infected cells reflected differences in haemagglutinin (HA) receptor binding affinity [24].

Bcl-2 and Bcl-xL are well-known targets of the proapoptotic protein Bcl-2 antagonist of cell death (BAD), which specifically blocks the activity of both antiapoptotic factors z by forming heterodimeric complexes with either of the two proteins and displacing Bax [15–26].

One of its downstream targets is the I*κ*B kinase (IKK), which once engaged leads to the activation of the transcription factor NF-*κ*B. In fact, under resting conditions, NF-*κ*B is sequestered in the cytoplasm through interaction with I*κ*B protein that prevents NF-*κ*B from entering the nucleus [27].

NF-*κ*B is sensitive proinflammatory miRNA with a relatively short half-life of about 2 h in the human CNS [23, 28].

Following NF-*κ*B activating stimuli, IKK is activated and phosphorylates I*κ*B, thus promoting nuclear translocation of NF-*κ*B to induce transcriptional activation of survival proteins, including Bcl-XL, Bcl-2, and X-linked inhibitor of apoptosis protein (XIAP) [29, 30].

Importantly NF-*κ*B activation is usually terminated via I*κ*B protein resynthesis and NF-*κ*B reinhibition [28–32].

From the multiple domains of NS1 of influenza A virus (NS1A) only one, the N terminal binding double stranded RNA domain, has been shown to be shared with the NS1 protein of influenza B virus (NS1B) [33].

Considering that the manipulation of apoptosis induction during influenza virus infection is partly associated with a motif of NS1A protein, with no homologous function in NS1B protein, we proposed the apoptotic pathways that could be differently affected by influenza A (both strains) and B viruses.

## 2. Materials and Methods

### 2.1. Cells

The IFN-competent human cells include alveolar epithelial cell line A549 and were obtained from central public health laboratories in Egypt (CPHL), Madin-Darby canine kidney cells (MDCK II), European Collection of Cell Cultures (ECACC), London, UK, recommended by World Health Organization for influenza virus replication and were obtained from central public health laboratories in Egypt (CPHL), and were kept in maintenance medium containing Dulbecco's Modified Eagle Medium, (DMEM; Gibco, Paisley, UK), 2 mM l-Glutamine (Gibco), 1× NEAA (Nonessential Amino Acids, Gibco), and 24 mM HEPES (N-2-hydroxyethylpiperazine-N-2-ethane sulfonic acid, Gibco) at 30°C. MDCK II cells were subcultured at 3-4 × 10^4^ cells/cm^2^ with growth medium maintenance medium supplemented with 10% fetal bovine serum (FBS; Gibco). Growth medium was supplemented with 1 mg/mL Geneticin® (Gibco) for cell line continuation or with 2.5 *µ*g/mL Fungizone® (Gibco) and 1× PSN (Penicillin-Streptomycin-Neomycin Antibiotic Mixture, Gibco) when cells were plated to be infected. Cultures were incubated at 37°C for 48 h prior to infection to reach a minimum of 90% monolayer confluence.

### 2.2. Viruses

Influenza A (subtype A (H1N1) pdm09) (isolated in CPHL in Egypt) and influenza A (subtype A (H3N2) isolated in CPHL in Egypt) and influenza B/Yamgata isolated in CPHL in Egypt viruses were propagated on MDCK II cells for stock constitution. Briefly, MDCK II cells in monolayer were infected with the virus and left to stand for 30 min for virus adsorption as controls.

Monolayer of the cells at a concentration of 1 × 10^6^ cells/mL was infected with the virus at a multiplicity of infection (MOI) of 0.1 and 1.0 PFU/cell in the presence of supplemental trypsin. Following adsorption for 1 h at 37°C, the inoculum was removed and washed before DMEM replaced.

The cultures were incubated up to 48 hours postinfection (hpi) and observed by inverted light microscopy for cytopathic effect (CPE). For each cell, four different sets of tissue culture flask were infected. Mock infected cells served were titrated before and after infection as described previously [34].

### 2.3. Cell Viability Assay

A549 and MDCK cells were infected by H3N2, H1N1 PDM 09, and influenza B virus at MOI of 0.1 and 1.0 PFU/cell and culture supernatants obtained at 8 h intervals up to 48 hpi. Cell viability following viral infection was determined by 3-(4, 5-dimethylthiazol-2-yl)-2, 5-diphenyl tetrazolium bromide (MTT) assay.

In this case, each cell was seeded in 96-well culture plates (approximately 5,000 cells per well) incubated with the related culture supernatants. The cells were washed with PBS and incubated with DMEM and 50 *µ*L/well of MTT solution (5 mg/mL) for another 3 h at 37°C. Then, the medium was totally removed, and 200 *µ*L of 0.04 N HCl in isopropanol was added to each well, and the plate was incubated for 1 h at room temperature. Optical density value was measured at 540 nm using an ELISA reader.

Cell survival was expressed as the ratio of virus infected to uninfected control. The three independent experiments were performed.

### 2.4. LDH Assay

The media of infected cells were removed from each condition at 8, 24, 36, and 48 h postinfection (hpi) and centrifuged at 250 ×g for 4 min. Total cell death was determined by measuring the release of LDH from cells with the CytoTox96® Nonradioactive Cytotoxicity Assay kit (Promega, Madison, IL, USA), according to the manufacturer's instructions. The cell monolayer was recovered for morphological analyses of apoptosis [35].

### 2.5. DNA Fragmentation

Cell death was evaluated by fragmentation of genomic DNA. Samples of virus infected cells were centrifuged, and the cell pellet was resuspended in 300 mL of cold cell lysis buffer (10 mM Tris, 0.5% Triton X-100 (pH 7.5)) and then incubated on ice for 30 min. The lysates were centrifuged at 12,000 rpm for 10 min at 4°C, and the supernatants were extracted once with buffered phenol and once with chloroform.

The DNA was precipitated with 300 mM NaCl and ethanol. DNA samples were resuspended in 50 *μ*L of Tris-EDTA buffer (10 mM Tris, 1 mM EDTA (pH 7.5)) treated with RNase A. The extracted DNAs were electrophoresed through a 2% agarose gel and stained with ethidium bromide.

### 2.6. Fluorescent Microscopy

The mock and infected cells were washed twice with PBS and fixed with 4% paraformaldehyde before being stained with anti-mouse IgG: FITC conjugate to each (1 mg/mL) for 30 min at 37°C. Stained cells were observed with a fluorescence microscope (Olympus BX51).

### 2.7. Real-Time RT PCR Analysis

Total RNA was extracted from cells using Qiagen according to the manufacturer's protocol. Five hundred nanograms of purified mRNA was used to generate cDNA with random hexamer primers (Thermo Scientific) and Revert Aid H Minus M-MuL V Reverse Transcriptase (Thermo Scientific) according to the manufacturer's protocol.

The quantitative real-time PCR (qRT-PCR) reaction mixture (25 *µ*L) consisted of the following: 12.5 *µ*L of Maxima SYBR green PCR master mix (Thermo Scientific), 0.5 *µ*L of cDNA template, and 1 *µ*L of each primer (100 *µ*M forward and reverse primers) (Table 1). Reactions were run in duplicate on Applied Biosystems 7500 real-time PCR system. The cycling conditions were as follows: 2 min at 50°C, 2 min at 95°C, and 50 cycles, with 1 cycle consisting of 15 s at 95°C and 30 s at 60°C. Threshold cycle (Ct) values were normalized to the values for the GAPDH control and compared with *β*-actin controls primers for real-time PCR of RNA transcripts.

## 3. Results

**Figure 1:**
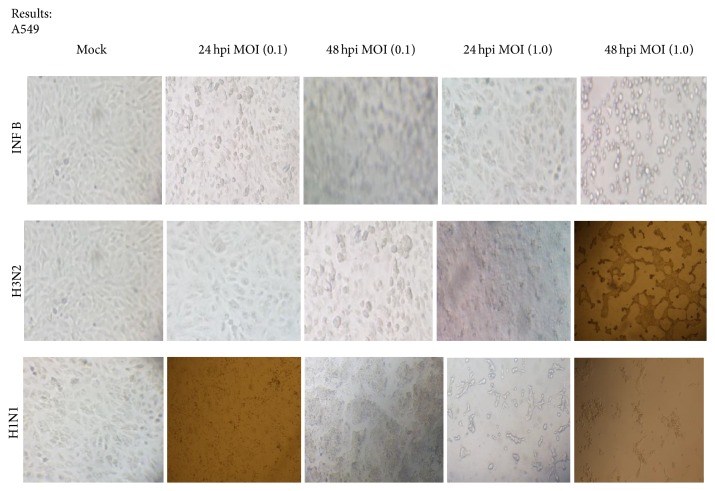
Cytopathogenicity of A549 cells to an influenza A and B viruses infection at 24 and 48 hours after infection (10x magnification).

To study the cytopathogenicity effect of the studied cells to Flu A/Pdm H1N1 09, Flu A/H3N2, and Flu B/Yamagata viruses, A549 and MDCK II cells were infected at MOI 0.1 and 1.0, although the marked CPEs were evident at 16 hpi for A549 cells infected with Flu B and H1N1 at higher MOI 1.0 which increased with time. At 24 hpi approximately half of the infected cells appeared smaller and irregularly shaped compared to mock cells. All studied influenza virus strains did not affect the CPE at MOI 0.1 on A549 cells (Figure 1). Infection of MDCK II cells with Flu B and H1N1 viruses caused a fast CPE especially at MOI 1.0 and a rapid fall in pH of cell suspension.

The granular and fragmented cells became obvious within 24 to 48 hpi. The infectious viruses were titrated by plaque assay at 16, 24, and 48 hpi. Higher viral titers were observed in infected A549 cells, and the peak of titer reached 48 hpi; however, statistical differences were not observed between MDCK II and A549 cells at MOI 1.0. The mean peak titers of the INF B virus in these cells reached 4.3 and 4.4 PFU/mL, respectively. The mean peak titers of the H3N2 virus in these cells reached 2.0 and 2.1 PFU/mL, respectively, until 48 hpi at both MOIs.

In addition, development of countable plaques required more days of incubation for H3N2 compared to other viruses.

The detection of lactate dehydrogenase (LDH) can be used to evaluate efficiently anti-influenza viruses' agents. LDH levels in the virus infected MDCK II and A549 cells were significantly higher than in controls, were in proportion to the degree of virus infection, and corresponded to a decrease in mitochondrial dehydrogenase activity.

LDH cytotoxicity assays were performed with different MOI of H3N2, Flu B, and H1N1 viruses at 8 h interval exposure times in A549 and MDCK II cells (Figures 4(a) and 4(b)).

Induction of general cell death in Flu A/Pdm H1N1 09, Flu A/H3N2, and Flu B/Yamagata infection differs in time and intensity. While cell death induced by INF B occurred earlier in infection, at 24 h postinfection (hpi) (*p* < 0.05), compared to H1N1 and H3N2 infection mediated cell death that occurs after 32 hpi (Figures 4(a) and 4(b)) in both cell lines. The infected A549 and MDCK II cells at higher MOI showed significantly cell death confirming the DNA fragmentation and nuclear condensation results. Regarding intensity of cell death induced by infection, H1N1 was shown to be more virulent, reaching a maximum of 4-fold increased cell death at 48 hpi in MDCK II cells and up to 5-fold in A549 cells, compared with mock infection (*p* < 0.01); exposure to INF B virus was shown to be less toxic to cells, and the cell viability was decreased with the increase in virus MOI in A549 and MDCK II cells, compared with mock and infected cells at MOI 1.0. Thus, these results suggest that LDH release and apoptosis start earlier, but with lower intensity, in cells infected with INF B than with H1N1 and H3N2, respectively (Figures 4(a) and 4(b)). A detectable DNA laddering in the virus infected cells appeared by 16 hpi at MOI 1.0 (Figure 3(b)). This pattern significantly progressed at 48 hpi.

The INF B infected A549 cells demonstrated nuclear condensation, which can be detected by FITC staining, as shown in Figure 3(a).

**Figure 2:**
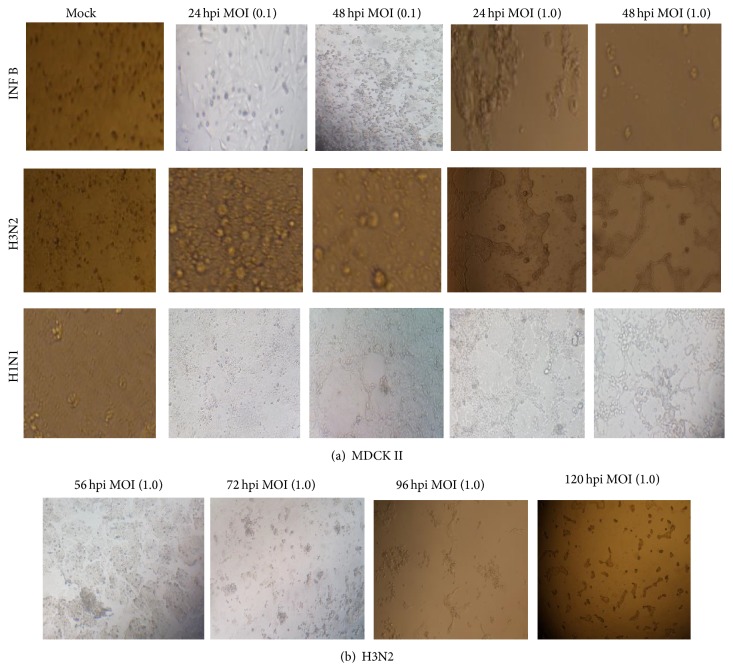
(a) Cytopathogenicity of MDCK II cells to an influenza A and B viruses infection at 24 and 48 hours after infection. (b) The delayed onset of cytopathogenicity by H3N2 (10x magnification).

**Figure 3:**
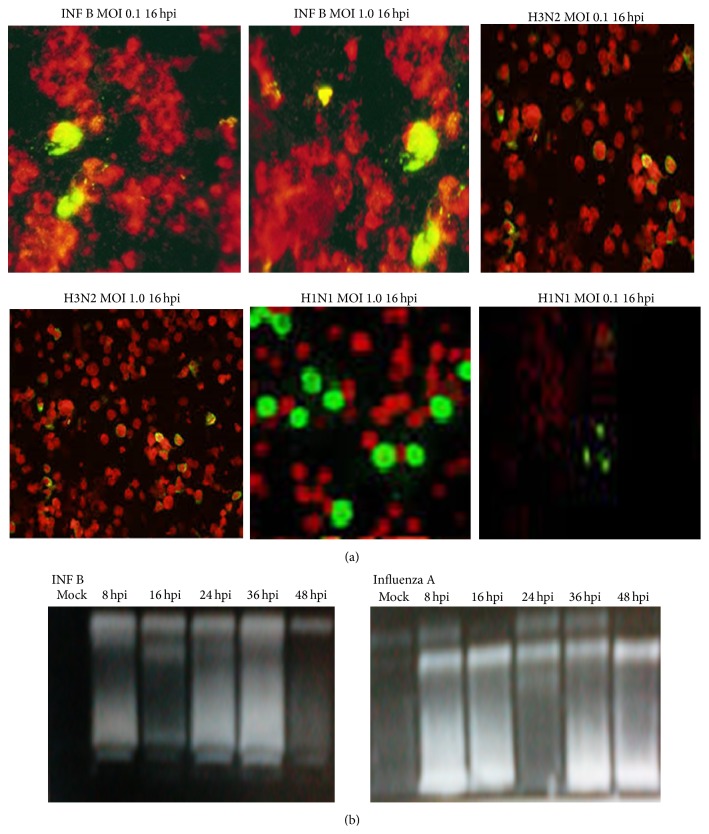
Influenza A and b viruses induce apoptosis in A549 and MDCK II cells at MOI 1.0. (a) Nuclear staining of the infected cells with FITC staining. (b) Chromosomal DNA fragmentation. DNAs were prepared from A549 cells infected with virus at 8 h intervals and separated by 2% agarose gel electrophoresis, followed by staining with ethidium bromide.

The infected A549 cells undergoing apoptosis exhibited some cells with fragmented nuclei. The virus mainly caused cell death in MDCK II cells; therefore, it seems that H3N2, INF B, and H1N1 virus induced apoptosis in A549 cells in dose and time dependent manner (Figures 1 and 2(a)).

To determine whether the death receptor or mitochondrial pathways modulated apoptosis, samples from the infected cells were analyzed for the levels of expressions of proapoptotic marker bad and caspase 3, caspase 8, and caspase 9 using quantitative RT-PCR (Table 1).

The presence of a significance increase in the tested markers compared to mock and nonapoptotic cells indicating low level or undetectable tested markers in these cells (Tables 2, 3, and 4).

A dose and time dependent activation of caspase 3, caspase 8, and caspase 9 proteins was observed in H3N2, INF B, and H1N1 infected A549 and MDCK II cells (Tables 2, 3, and 4).

The levels of caspase 8 protein activation were not detected in INF B infected MDCK II cells (Table 3); however, the cell viability was significantly lower in MDCK II than A549-infected cells (Figure 2).

The apoptotic markers in A549 and MDCK II cells were evaluated by quantitative RT-PCR (Table 1).

However, different regulation levels of MxA mRNA were observed in A549 and MDCK II cells infected by H1N1, H3N2, and INF B, and expression of MxA gene was upregulated at 16 hpi which reached to maximal level at 24 hpi compared with mock cells; however, the higher dose of virus is, a weaker MxA expression was detected (Tables 2, 3, and 4).

MxA expression showed similar pattern of induction peaking on 24 hpi and decreasing by 48 hpi in MDCK II cells at both infectious doses in all studied influenza subtypes (Tables 2, 3, and 4).

MxA mRNA expression for A549 cells was very high in response to INF B infection compared to H1N1 and H3N2 infection. However, it is same order in MDCK II infected cell but with lower response than A549 infected cell (Tables 2, 3, and 4).

### 3.1. The Delayed Onset of Cytopathogenicity by H3N2

Influenza H3N2 virus infected A549 cells also elicited less TNF alpha and FasR transcription than either INF B or H1N1. These observations can account for the lower apoptotic response in influenza H3N2 virus infected lung (Figure 2(b)). As little impact on the expression of intrinsic pathway components was observed, it seems that the apoptotic response to influenza virus infection in A549 cells was mainly through the extrinsic pathways (Table 2).

The delayed onset of cytopathogenicity by H3N2 may take more than 96 hpi to obtain clear cytopathogenic effect compared to INF B or H1N1 Figure 2(b).

In addition, the pattern observed for general cell death seems to correlate with that observed for apoptosis, in all studied influenza subtypes.

To evaluate potential differences of apoptotic signaling pathways triggered by influenza A and B viruses infection, we measured the activity of several caspases, such as the effector caspase 3, the extrinsic pathway associated initiator caspase 8, and the intrinsic pathway associated initiator caspase 9.

Results show that H1N1 and H3N2 infection induced caspase 3 activities from 16 to 48 hpi, respectively (Tables 2 and 4). In addition, the activity of effector caspases increased from 16 hpi with INF B (Table 3), remaining higher than H1N1 and H3N2, respectively, infected cells, and it is seen clear in A549 cells than MDCK II cells, until 48 hpi. In fact, this early effect of INF B in inducing the activity of effector caspases, when compared with H1N1 and H3N2, respectively, corroborates the previously observed apoptotic patterns (Tables 2, 3, and 4).

The differences between H1N1 and H3N2, respectively, and INF B virus mediated caspase activation were also evident for caspase 9 and caspase 8 (Tables 2, 3, and 4).

The activity of caspase 9 slightly increased in H1N1 and H3N2 infected cells, respectively, during the first 48 hpi, when compared with mock infection (Tables 2 and 4). In contrast, INF B induced lower levels of caspase 9 activity during the first 24 hpi although persisting until 48 hpi, in MDCK II cell lines and undetectable in A549 cells (Table 3). Regarding caspase 8, our data proved that activation of this death receptor related caspase was detected as early as 16 hpi after INF B infection in A549 cells, where less efficiency can be detected on MDCK II cells, becoming only evident at later stages of H1N1 and H3N2 infection, respectively (Tables 2, 3, and 4).

Therefore, these results indicate that influenza H1N1 and H3N2 and INF B viruses infection mediate different apoptosis profiles.

**Figure 4:**
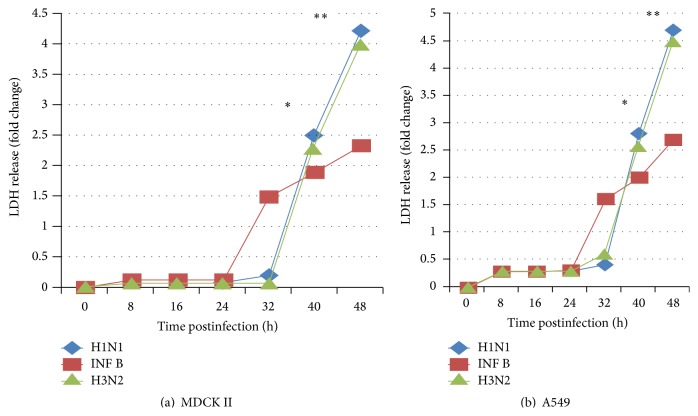
Flu A/Pdm H1N1 09, Flu A/H3N2, and Flu B/Yamagata viruses induce cell death and apoptosis profiles. Effects of influenza infection (MOI 1.0) on viability of MDCK II (a), and A549 cells (b) were determined by LDH assay. The *∗* indicates significant differences (*p* < 0.05) and ^*∗∗*^ *p* < 0.01 from the mock cells at the same time after infection.

INF B induces an immediate apoptotic response, but INF B induced cell death levels are depending on the type of cells compared to those induced by H1N1 and H3N2, respectively (Figure 4). In addition, although all virus strains induced extrinsic and intrinsic apoptosis, H1N1 and H3N2, respectively, prefer to initially enhance the intrinsic pathway, as determined by caspase 9 activities and INF B prefer intrinsic pathway according to caspase 8 activity in A549 cell line but also can choose extrinsic pathway as determined by caspase 9 activity in MDCK II cells.

### 3.2. Influenza A and B Viruses Differentially Regulate NF-*κ*B Activation

One of the important signaling factors for virus replication is the transcription factor NF-*κ*B, also commonly regarded as a major regulator of the innate immune defense to infection. In fact, NF-*κ*B is often activated by viruses, leading to the upregulation of a variety of antiviral genes. Several molecular mechanisms have been identified to confer this virus-supportive function of NF-*κ*B. First, it was shown that NF-*κ*B acts via induction of proapoptotic factors, such as TNF-related apoptosis inducing ligand (TRAIL) or FasL, and subsequent activation of caspases [36].

To address whether differences in apoptosis profiles between influenza A and B strains depend on NF-*κ*B factor, the levels of NF-*κ*B versus its endogenous inhibitor, the I*κ*B (inhibitor of kappa B), and tumor necrosis factor alpha (TNF*α*) which induced NF-*κ*B activation [37] were evaluated throughout H1N1 and H3N2 and INF B virus infection, respectively, and compared between all strains (Tables 2, 3, and 4). The analysis of the TNF*α* and I*κ*B protein levels revealed that infection with INF B induced TNF*α* and I*κ*B ratio earlier at 16 hpi, higher than that observed in mock infection in both cell lines (Tables 2, 3, and 4).

Regarding H1N1 and H3N2 infection, respectively, although the maximum value for TNF*α* and I*κ*B ratio was also observed at 16 and 48 hpi, it remained always higher than that detected for H1N1 and H3N2, respectively, at 16 and 48 hpi, or for mock infection, during 16 and 48 hpi. This may be explained by the continuous increase of I*κ*B*α* protein levels throughout INF B infection (Tables 2, 3 and 4). To confirm whether influenza A or influenza B strains mediated increase in TNF*α*/I*κ*B ratio reflects the enhancement in NF-*κ*B activity, the transcriptional activity of NF-*κ*B was studied throughout H1N1 and H3N2 and INF B and mock infection periods. From the upper data, these results revealed that NF-*κ*B survival pathway activation might be the responsible factor for the distinct apoptosis profiles induced by H1N1 and H3N2 and INF B infection. Curiously, in this cellular context, NF-*κ*B might be activated by mechanisms dependent on the regulation of I*κ*B protein levels.

## 4. Discussion

Viruses have evolved multiple and complex strategies to subvert and evade the host immune-response to ensure their own replication and survival [31, 38, 39].

Although the death receptor mediated apoptosis pathway induced by influenza infection has been extensively studied [40–42], little is known about the differences in the kinetics and pathways of apoptosis induction in influenza A and B viruses infections. Early in infection, viral NS1 binds to the PI3K subunit p85 and activates the kinase [23].

This results in activation of Akt/PKB via phosphorylation by pyruvate dehydrogenase kinase and the mammalian target of rapamycin complex 2 [43].

Activated Akt negatively regulates proapoptotic factors [caspase 3, caspase 9, Bcl-2 associated death promoter (BAD), and GSK-3] and thereby suppresses early apoptosis [44].

At later stages of infection, the virus-induced NF-*κ*B activation leads to the expression of proapoptotic factors (Fas, FasL, and TRAIL), thereby, to caspase induction, resulting in increased RNP export [28].

During productive virus infection, the proapoptotic factors TRAIL and FasL are expressed in an NF-*κ*B-dependent manner.

The classical mechanism of NF-*κ*B activation includes activation of IKK, which phosphorylates the inhibitor of NF-*κ*B and I*κ*B and targets the protein for subsequent degradation. This leads to the release and migration of the transcriptionally active NF-*κ*B factors, such as p65 or p50, to the nucleus [43].

The early induction of extrinsic pathway of apoptosis by influenza B infection, detected by the increase in the activity of the initiator caspase 8, reveals the inability of influenza B strain to avoid cell death through this pathway.

Curiously, the extrinsic pathway of apoptosis has been previously associated with the innate antiviral cell response that leads to the production of interferon (IFN) *α*, IFN *β*, and cytokines capable of inducing apoptosis by the TNF-related apoptosis ligand (TRAIL) pathway, which in turn trigger the extrinsic pathway of apoptosis by the cell death receptor pathway [17, 45].

In contrast, the induction of the extrinsic apoptosis pathway was only observed for influenza A infection at 16 hpi, reflecting the ability of the influenza A virus to counteract with this antiviral cell response.

The PI3K/Akt pathway, one of the pathways hijacked by the NS1 protein of influenza A virus, was already shown to be responsible for a delay in induction of apoptosis in the host cells [46].

The PI3K/Akt pathway was activated in a similar extent by the influenza A and B viruses strain infection. However, influenza A strain was able to induce a second activation of this pathway later throughout the infection period (24 hpi).

Although the initial activation of this pathway by influenza A strain could partially explain the delay in apoptosis induction of influenza A in comparison with influenza B, additional mechanisms must be in place by influenza A in infected cells to block preactivated apoptosis and avoid cell loss [3, 5].

We have provided experimental evidences that influenza virus H3N2 induces an apoptotic response mediated by caspases activation for the host cells that is similar to but delayed compared with that induced by human influenza viruses including H1N1 and INF B.

Activated caspase 8 is able to cleave additional downstream caspases and also activate Bid; proapoptotic member of the Bcl-2 proteins resulted in an efflux of cytochrome c into the cytoplasm [47].

The viral infection at higher MOI induced cleavage of procaspase 8 into intermediate p41–43 at the earlier stage of infection (24 hpi) followed by activation to its p18 active at 48 hpi using an antibody directed against the N terminal fragment of this protein [34].

NF-*κ*B activation has already been associated with the antiviral response of the influenza A infected cells [45, 48].

It is possible that NF-*κ*B may also be required for influenza A virus replication by activating the transcription of antiapoptotic factors [4, 49].

In fact, when comparing with influenza B strain, influenza A strain infection markedly increased the levels of the NF-*κ*B downstream survival targets, including Bcl-XL and XIAP. However, the influenza A mediated effects were only associated with a delay in apoptosis induction, rather than within crease in apoptotic resistance of infected cells.

Although the differences observed in kinetics and pathways of induction of apoptosis between influenza A and B strain infections point to occur at the I*κ*B/NF-*κ*B level, further studies are required to clarify the molecular mechanisms by which influenza B virus induces I*κ*B accumulation.

A better understanding of the molecular mechanisms by which influenza A and B viruses regulate apoptosis in host cells have also the potential to uncover the networks of factors that may contribute to a successful influenza virus replication, crucial for the development of vaccines and antiviral drugs [5, 28].

### 4.1. Influenza A and B Viruses Differentially Modulate the Expression of I*κ*B Protein Level

To analyze the differences on apoptosis regulatory mechanisms between H1N1 and H3N2 and INF B infection, namely, the involvement of I*κ*B protein level activation in these events, the expression levels of survival downstream targets of I*κ*B protein level, including proapoptotic BAD levels, were studied throughout the infection periods.

RT-PCR showed that BAD levels are highly pronounced in INF B than in H1N1 and H3N2, respectively. In fact, at 16 hpi, INF B showed higher levels of BAD when compared with mock infection. The levels of BAD were expressed in lower levels when compared with those observed after 16 h of H1N1 and H3N2 infection. These findings suggest distinct regulatory networks of apoptosis between H1N1 and H3N2 and INF B infection, which might support the role of the prosurvival factor NF-*κ*B and its target genes in delaying apoptosis initiation and CPE in H3N2 infection compared with influenza B and H1N1 infection, respectively.

Influenza virus-induced cytopathic effect (CPE) and cell death are inhibited in BAD deficient cells. Influenza virus killing of host cells is known to occur through the activation of the apoptotic signaling pathway [50, 51].

BAD is an important regulator of antiapoptotic Bcl-2 and Bcl-xL proteins. Its blockage of Bcl-2 and Bcl-xL defines the fate of the host cell toward apoptosis [51].

Infected cells were examined visually for demonstration of cytopathic effect (CPE), which phenotypically manifests as rounding up and detachment of infected cells as well as abnormal cellular structural morphology BAD knockdown in A549 cells reduced influenza virus replication of different virus subtypes.

A number of studies have reported the important role apoptosis plays in promoting efficient influenza virus replication [39, 42].

The delayed onset of apoptosis by H3N2 influenza virus infected respiratory epithelial cells A549 may be a mechanism for the influenza viruses to have more prolonged replication within the human respiratory tract, and this may contribute to the pathogenesis of human disease [52].

In contrast to [42] the delayed CPE in the H1N2 infected cells might result from decrease of cell death rate, cytochrome c release, and apoptosome formation as compared to those of H1N1 and H3N2 infected cells.

Our study also showed that the considerable MxA expression was found in influenza A and B virus and infected A549 and MDCK II cells at low dose of the virus.

MxA mRNAs were induced in the infected A549 cells in a dose dependent manner, correlating with the replication of influenza A and B viruses in the cell. Activation of the cellular antiviral defense by MxA expression decreases virus replication at early time of infection which had not significant impact on final virus titers. The result suggests a role for IFN response in the replication of influenza A and B viruses that may provide some degree of host resistance in the early stages of infection. In contrast to MDCK II, the A549 cells are IFN-competent cells, and MxA is upregulated in these cells while the protein is either downregulated or even undetectable in MDCK II cell in response to influenza A and B infection. Thus, an insufficient antiviral defense in MDCK II cells promotes efficient influenza A and B viruses replication which is controlled in part by the IFN response. Taken together, the results demonstrated that the permissively of noncompeting IFN MDCK II cells for influenza A and B viruses is comparable with A549 cells; however, MDCK II cells are not target tissue that respond to the infection. These data revealed that the sensitivity of human alveolar epithelial cells to influenza A and B viruses induced apoptosis signaling via intrinsic and extrinsic pathways in parallel, and the induction was associated with viral infection in a dose dependent manner.

In conclusion, influenza viruses afflict millions of people each year and cause serious medical complications. Because of the high genetic variability of influenza viruses, the development of effective vaccines against pandemic influenza is still an ongoing challenge. In addition, although all H1N1 and H3N2, respectively, prefer initially to enhance the intrinsic pathway, as determined by higher caspase 9 activity in MDCK II cells compared to caspase 8 activity and vice versa in A549 cells at different MOI, INF B prefer extrinsic pathway according to significantly low or undetectable caspase 9 activity and high activity of caspase 8 in A549 cell line but also can choose intrinsic pathway as determined by significant low or undetectable caspase 8 activity and high caspase 9 activity in MDCK II cells at different MOI.

## Figures and Tables

**Table 1:** 

Name	Direction	Sequence 5′→3′
MxAF	Forward primer	TTCAGCACCTGATGGCCTATC
MxAR	Reverse primer	TGGATGATCAAAGGGATGTGG
b-actin	Forward primer	GAG ACC TTC AAC ACC CCG C
b-actin	Reverse primer	ATG TCA CGC ACG ATT TCC C
BAD	Forward primer	ACCCGGCAGACAGATGAG
BAD	Reverse primer	CTTCCTCTCCCACCGTAGC
I*κ*B	Forward primer	CAGCAGACTCCACTCCACTT
I*κ*B	Reverse primer	GAGAGGGGTATTTCCTCGAA
TNF-*α*	Forward primer	CTCTTCTCCTTCCTGATCGTGGCA
TNF-*α*	Reverse primer	GTTGGATGTTCGTCCTCCTCACA
Caspase 3	Forward primer	TTAATAAAGGTATCCATGGAGAACACT
Caspase 3	Reverse primer	TTAGTGATAAAAATAGAGTTCTTTTGTGAG
Caspase 9	Forward primer	AGCCAGATGCTGTCCCATAC
Caspase 9	Reverse primer	CAGGAGACAAAACCTGGGA
Caspase 8	Forward primer	CTGGGAAGGATCGACGATTA
Caspase 8	Reverse primer	CATGTCCTGCATTTTGATGG
GAPDH	Forward primer	GGCATTGCTCTCAATGACAA
GAPDH	Reverse primer	TGTGAGGGAGATGCTCAGTG

**Table 2:** Viral and cellular genes expression levels in response to H3N2 influenza virus of A549 and MDCK cells evaluated by quantitative RT-PCR at the indicated time point. Ct values were determined for caspases 3, 8, and 9, TNF*α*, I*κ*B*α*, BAD, MxA, and *β*-actin and GPDH genes.

H3N2	I*κ*B*α*		BAD		TNF		Caspase 9		Caspase 8		Caspase 3		MXA		*β*-actin		GPDH	
	MOI 0.1	MOI 1.0	MOI 0.1	MOI 1.0	MOI 0.1	MOI 1.0	MOI 0.1	MOI 1.0	MOI 0.1	MOI 1.0	MOI 0.1	MOI 1.0	MOI 0.1	MOI 1.0	MOI 0.1	MOI 1.0	MOI 0.1	MOI 1.0
MDCK																		
16 hpi	22.46	22.76	16.38	15.85	25	25.8	24.2	25.3	25	26	26	276	27	26	17.2	17.2	22.2	22.2
24 hpi	26.57	26.33	19.77	20.98	28.4	28.79	27.6	28.4	29	30	31.2	30.8	28	29	17.5	17.6	23.6	23.6
36 hpi	24.82	25.2	18.4	19.9	26.72	26.99	26.4	27.0	27	27	28.7	27.4	26	28	17.9	18	23.2	23.2
48 hpi	23.2	23.22	24.66	27.75	25.9	25.9	25.69	27	26	26	26.9	26.4	26	26	18.3	18.4	23	23
A549																		
16 hpi	19.2	19.69	16.8	18.4	20	20.9	22.8	21.9	19	20.6	21	21.6	27	27	16.9	16.9	20.3	20.6
24 hpi	22.63	22.75	17.55	17.66	23	22.7	23	22.7	23.2	24.1	25.2	25.2	31	31.66	17.2	17.2	21.0	21.0
36 hpi	21.25	22.25	18.22	22.5	24	22.9	24	23.6	21.65	21.45	23	23	29.4	29.88	17.9	17.8	21.0	21.0
48 hpi	19.36	20.15	17.2	17.95	22	21.4	21.77	22.2	21	19.8	21.9	21.88	27.4	27.89	17.2	17.0	21.0	21.0

**Table 3:** Viral and cellular genes expression levels in response to influenza B virus of A549 and MDCK cells evaluated by quantitative RT-PCR at the indicated time point. Ct values were determined for caspases 3, 8, and 9, TNF*α*, I*κ*B*α*, BAD, MxA, and *β*-actin, and GPDH genes.

INF B	I*κ*B*α*		BAD		TNF		Caspase 9		Caspase 8		Caspase 3		MXA		*β* actin		GPDH	
	MOI 0.1	MOI 1.0	MOI 0.1	MOI 1.0	MOI 0.1	MOI 1.0	MOI 0.1	MOI 1.0	MOI 0.1	MOI 1.0	MOI 0.1	MOI 1.0	MOI 0.1	MOI 1.0	MOI 0.1	MOI 1.0	MOI 0.1	MOI 1.0
MDCK																		
16 hpi	16	16.76	18.38	18.85	20	20.4	23.2	24.3	—^*∗*^	—^*∗*^	16.8	16.6	22	23	17.2	17.2	22	22.2
24 hpi	20.57	20.33	22.77	22.9	24.63	24.89	26.6	27.4	—^*∗*^	—^*∗*^	21.2	20.8	26	26.7	17.6	17.6	23.5	23.6
36 hpi	18.82	18.63	20.4	20.9	22.78	23.16	25.4	26.0	—^*∗*^	—^*∗*^	18.7	17.4	24.2	24.5	18	18	23.0	23.2
48 hpi	17.2	17.22	19.66	19.75	20.9	20.9	24.6	25.9	—^*∗*^	—^*∗*^	16.9	17.4	22.7	22.9	18.4	18.4	23.1	23
A549																		
16 hpi	18.2	18.44	19.8	18.4	19.4	19.9	—^*∗*^	—^*∗*^	19.8	20.6	15.9	15.9	19	27	16.9	16.9	21	20.66
24 hpi	22.63	22.75	23.55	24.1	22	21.7	—^*∗*^	—^*∗*^	22.2	23.1	19.2	19.2	22	22.7	17.4	17.2	21	21.0
36 hpi	20.25	20.6	21.2	21.7	21.2	21.9	—^*∗*^	—^*∗*^	21.65	21.45	17	17	20.4	20.8	17.9	17.8	21.3	21.0
48 hpi	18.9	19.15	20	20	20.7	20.2	—^*∗*^	—^*∗*^	21.4	19.6	16.9	16.4	19.4	19.6	17.2	17.0	21.1	21.0

^*∗*^Undetectable or very low expression level.

**Table 4:** Viral and cellular genes expression levels in response to H1N1 influenza virus of A549 and MDCK cells evaluated by quantitative RT-PCR at the indicated time point. Ct values were determined for caspases 3, 8, and 9, TNF*α*, I*κ*B*α*, BAD, MxA, and *β*-actin and GPDH genes.

H1N1	I*κ*B*α*		BAD		TNF		Caspase 9		Caspase 8		Caspase 3		MXA		*β*-actin		GPDH	
	MOI 0.1	MOI 1.0	MOI 0.1	MOI 1.0	MOI 0.1	MOI 1.0	MOI 0.1	MOI 1.0	MOI 0.1	MOI 1.0	MOI 0.1	MOI 1.0	MOI 0.1	MOI 1.0	MOI 0.1	MOI 1.0	MOI 0.1	MOI 1.0
MDCK																		
16 hpi	18.9	20	21.6	21.8	22	22	20	20	22.6	23	22	22	16.5	16.9	17.2	17.2	22.2	22.2
24 hpi	21	21	23.8	24.2	24	24.9	25.4	24.6	25.2	24.6	26	26	20	20.8	17.6	17.6	23.6	23.6
36 hpi	20	19	21.8	22.2	22.6	22.4	23	23.6	23	23	24	24.4	18.4	18.6	18	18	23.2	23.2
48 hpi	18.9	18.6	21.75	21.9	22.9	22.88	21.3	21.6	22.9	23.3	23	23.9	18.0	21.7	18.4	18.4	23	23
A549																		
16 hpi	19.6	18.7	16.82	17.1	20.7	20.88	21.6	21.2	19.4	19.8	18	17	18.2	29.4	17	16.9	20.7	20.66
24 hpi	23	23.8	19.4	19.6	21.89	22.25	26.7	26.8	22	23	20	20	21	21.3	17.2	17.2	21	21
36 hpi	22	21.98	18.3	18.7	21.2	21.3	24.0	24.2	21.8	21.9	19.2	19.8	20.4	20.6	17.8	17.8	21	21.0
48 hpi	20.45	19.2	17.66	18.2	20.9	20.9	21.81	21.8	20.8	20.8	17.6	18.2	19.2	19.8	17	17.0	21	21.0

## Abbreviations

Ct: Threshold level
CPE: Cytopathogenic effect
MOI: Multiplicity of infection
hpi: Hour postinfection
MDCK: Madin-Darby canine kidney cell.
